# Effects of Lenvatinib treatment for advanced differentiated thyroid cancer on cortisol deficiency

**DOI:** 10.3389/fendo.2025.1691740

**Published:** 2026-01-05

**Authors:** Salvatore Monti, Beatrice Fazzalari, Valerio Renzelli, Claudia Bongermino, Maria Francesca Lioni, Maria Grazia Deiana, Maurizio Poggi, Fedra Mori, Giuseppe Pugliese

**Affiliations:** 1 Endocrinology and Diabetes Unit, Azienda Ospedaliero-Universitaria Sant’Andrea, ‘‘Sapienza’’ University, Rome, Italy; 2 Clinical Trial Center (CTC), Azienda Ospedaliero-Universitaria Sant’Andrea, Rome, Italy

**Keywords:** adrenal insufficiency, fatigue, iodine-refractory differentiated thyroid cancer, Lenvatinib, thyroid cancer

## Abstract

**Background:**

Lenvatinib, a multi-kinase inhibitor widely used in the treatment of radioiodine-refractory differentiated thyroid carcinoma (RR-DTC), has shown remarkable efficacy and improvement in progression-free survival (PFS), although its use is associated with a variety of side effects. Among them, adrenal insufficiency (AI) remains under-recognized and potentially underestimated, and it may be involved in fatigue, one of the most frequent adverse events (AEs) Lenvatinib-related. In this prospective study, we report the incidence, development, and time course of primary AI (PAI) during Lenvatinib treatment in patients with RR-DTC followed at a single tertiary care center.

**Methods:**

The study was conducted on 39 consecutive patients with RR-DTC. Eight patients were excluded because they had a follow-up of less than 6 months or were receiving glucocorticoids for RR-DTC-related indications. We studied 31 patients selected for Lenvatinib therapy from June 2017 to December 2024. The median follow-up duration was 42.58 ± 29.53 months (range, 6–97 months). Performance status was evaluated for each patient using the Eastern Cooperative Oncology Group (ECOG) scale. Adrenal function was assessed by measuring serum cortisol and adrenocorticotropic hormone (ACTH) levels, and through the 250 μg ACTH stimulation test. Additionally, fatigue intensity was evaluated using the Common Terminology Criteria for Adverse Events (CTCAE) grading scale. Peak cortisol levels below 500 nmol/L (18.1 µg/dL) at 30 or 60 minutes after ACTH injection were indicative of adrenal insufficiency (AI) (PAI-18.1). A cutoff of 386.2 nmol/L (14 µg/dL) has also been used (PAI-14). In patients with primary adrenal insufficiency (PAI), steroid replacement therapy with cortisone acetate (CA) was initiated at doses ranging from 25 to 37.5 mg/day. Throughout follow-up, the ACTH stimulation test was repeated every 3 to 9 months, with a 72-hour discontinuation of CA prior to testing.

**Results:**

During Lenvatinib treatment, 24 of 31 patients (77.4%) developed primary AI (PAI)-18.1, and 14 of 31 patients (45.2%) developed PAI-14. Patients with a cortisol peak below 646.6 nmol/L at the initial ACTH stimulation test, prior to starting Lenvatinib, demonstrated a higher risk of developing both PAI-18.1 and PAI-14 during treatment. Patients who developed PAI during Lenvatinib treatment had significantly lower cortisol peak levels on the initial ACTH stimulation test performed before treatment initiation compared to those who did not develop PAI. Fatigue was observed in 28 of 31 patients (90%) during Lenvatinib treatment. Among patients who developed PAI, a significant improvement in fatigue was observed following initiation of CA therapy.

**Conclusions:**

Our findings suggest a higher occurrence of PAI, which may contribute to fatigue associated with Lenvatinib treatment. Routine adrenal function testing and early recognition of PAI are essential for timely diagnosis and effective glucocorticoid replacement therapy, enabling patients to continue Lenvatinib treatment with improved tolerability and adherence.

## Introduction

Differentiated thyroid carcinoma (DTC) represents approximately 95% of thyroid malignancies and is generally associated with a favorable prognosis.

Less than 10% of all patients develops distant metastases; 60-70% of them (nearly 5% of all thyroid cancer cases) will become radioiodine-refractory (RR-DTC), with a significant impact on the prognosis and a 5-year survival rate of around 10% (1).

Lenvatinib is an oral multi-target kinase inhibitor (MKI) targeting vascular endothelial growth factor receptors 1–3 (VEGFR1–3), fibroblast growth factor receptors 1–4 (FGFR-1–4), platelet-derived growth factor receptor α (PDGFRα), RET and KIT proto-oncogenes, used as a monotherapy for the treatment of progressive radioiodine-refractory differentiated thyroid cancer (RAI-R-DTC) which has allowed to increase the survival of these patients (2, 3).

On the other hand, Lenvatinib is also associated with several adverse events (AEs) including mainly hypertension, diarrhea, fatigue, anorexia, stomatitis, weight loss, palmar-plantar erythrodysesthesia syndrome and proteinuria. Among these listed, fatigue is one of the most frequent AE in patients receiving Lenvatinib leading to treatment reduction, discontinuation or withdrawal, with a possible reduction in efficacy and in progression free-survival (PFS) (4).

In recent years, it has emerged that primary adrenal insufficiency (PAI) may represent a contributing mechanism involved in the development of fatigue associated with Lenvatinib therapy (4, 5). However, this association has often been underestimated and misunderstood, likely due to the limited number of available studies, involving small sample sizes (5–9). In 2017, a patient receiving Lenvatinib at our Endocrine Center developed primary adrenal insufficiency (PAI) presenting with fatigue (6). Initiation of cortisone acetate (CA) replacement therapy led to a marked improvement in symptoms (6).

Therefore, in our center, adrenal function is evaluated performing the ACTH stimulation test (250 μg) in all patients before starting Lenvatinib and during follow-up.

## Materials and methods

We analyzed consecutive patients who started Lenvatinib treatment from June 2017 to December 2024.

All patients were affected by advanced progressive RR-DCT and have had tumor progression according to Response Evaluation Criteria in Solid Tumors (RECIST) 1.1 criteria (10) before beginning Lenvatinib therapy. Eligible patients had received no prior therapy with a tyrosine kinase inhibitor (TKI).

The study was performed at our Endocrine Center, a tertiary institution.

Written informed consent was obtained from all patients included in this study. The study was conducted in accordance with the ethical standards of the Institutional Research Committee and with the 1964 Helsinki Declaration and was approved by the Local Ethical Committee (CET Lazio Area 1, Rif. 8122).

For each patient, performance status (PS) was evaluated using the Eastern Cooperative Oncology Group (ECOG) scale.

Lenvatinib was started at dosages ranged from 4 to 24 mg according to patients’ clinical conditions and disease features. Drug-related adverse events (AEs) were assessed by National Cancer Institute Common Terminology Criteria for Adverse Events (CTCAE) version 5.0 (11) and Lenvatinib treatment was discontinued, interrupted or reduced according on AEs severity.

Patients with any history of adrenal disease, including adrenal metastases, nephrotic syndrome, liver disease, or who were on previous or current steroid treatment, or any drug (including estrogens) known to interfere with steroid hormone secretion and metabolism, were considered as non-eligible. Moreover, we included only patients treated with Lenvatinib for at least 6 months to evaluate development and time course of PAI, in accordance with our previous study (6).

All patients were evaluated before and after starting Lenvatinib treatment by anamnestic and physical examination, laboratory analyses and hormonal evaluation.

Patients underwent weekly clinical visits for the first month, followed by monthly clinical visits thereafter. Laboratory analyses were scheduled every month and adrenal function testing every three months.

Adrenal function was evaluated by cortisol and ACTH plasma and by ACTH (or cosyntropin) stimulation test. Blood samples for cortisol and ACTH baseline were collected fasting, between 8:00 and 9:00 a.m. Soon after baseline, every patient underwent intravenous (iv) injection of 250 μg ACTH (cosyntropin). Plasma cortisol was collected at 30 and 60 minutes after 250 μg ACTH stimulation test, according to current guidelines (12).

Peak cortisol levels below 500 nmol/L (18.1 mcg/dL) at 30 or 60 minutes indicate adrenal insufficiency (AI), according to current Endocrine Society Clinical Practice Guideline (12). However, more recently, considering or using the more specific (less cross-reactive) assays, a new serum cortisol cutoff of 14 to 15 μg/dL has been proposed to reduce false positive ACTH stimulation testing (13). For this reason, we used two cutoff values for the diagnosis of PAI: 500 nmol/L or 18.1 mcg/dL (PAI-18.1) and 386.2 nmol/L or 14 mcg/dL (PAI-14).

In patients with PAI, cortisone acetate (CA) replacement treatment was started. In any case, in these patients the ACTH stimulation test was repeated after at least three-six months, stopping CA for 72 hours before. Further ACTH stimulation was repeated after 6–12 months in those patients with sufficient follow-up.

CA replacement treatment was started with a dosage of 25-37.5 mg/day, according to weight and clinical conditions, in two divided doses per day. The highest dose was given in the morning at awakening and the next in the early afternoon, according to current guideline (12) and our previous experience (6).

All hormones were analyzed using a chemiluminescence immunoassay (CLIA). Sensitivity of assays was 1.13 pg/ml for ACTH and 0.5 nmol/L for serum cortisol; intra-assay and inter-assay coefficient of variations were 4.9 and 8.9% for ACTH and 4.3 and 5.5% for serum cortisol (LIASON XL Analyzer). Reference values were 4.7-48.8 pg/ml for ACTH and 101–536 nmol/L for cortisol.

### Statistical analysis

A descriptive analysis of all the sample parameters collected was carried out. The normality of the distribution of our continuous quantitative variables was verified through the Shapiro-Wilk test and confirmed by Kolmogorov-Smirnov test and D’Agostino & Pearson test. Continuous quantitative variables were reported as mean and standard deviation (SD). The qualitative variables were presented as absolute frequencies and percentages.

Statistical differences between two groups of continuous variables were assessed by paired or unpaired Student t test, when appropriate. Statistical differences between three or more groups of a continuous variable were assessed using Analysis of Variance (ANOVA). The comparison of frequencies between the groups of qualitative variables was analyzed by chi square or Fisher test, when appropriate. Correlation between different variables was determined with the Spearman test. The performance of cortisol peak after the cosyntropin test was evaluated by receiver operating characteristic (ROC) curve analysis and the most accurate cut-off calculated with sensitivity and specificity. A p<0.05 was considered as statistically significant and all tests were two-sided. All statistical analyses were performed by Graph Pad Prism 8 (Graph Pad Software Inc., USA).

## Results

From June 2017 to December 2024, 39 consecutive patients with advanced and progressive RR-DTC started Lenvatinib treatment at our Center; none of them received prior therapy with a tyrosine kinase inhibitor.

Eight patients were excluded from our study for the following reasons: 3 had a follow-up of less than 6 months; 5 were taking glucocorticoids for problems related to RR-DTC, but not for PAI.

The characteristics of enrolled patients are reported in **Table 1**. The thirteen patients reported in our previous study (5) are included in the present series. The mean age of the 31 patients enrolled was 68.03±10.15 (M±SD) years (range 40–86 years). Fifteen patients were female (48.4%) and 16 were male (51.6%) with a mean age of 66.9±9.19 (range 40-82) and 68.1±10.63 (range 44-86) years, respectively. The weight was 76.22±15.08 (M±SD) kilograms (Kg) (range 52-109.6 Kg). ECOG PS was 0 in 21 patients (67.7%), 1 in 9 patients (29%), and 2 in one patient (3.3%).

**Table 1:** Clinical and pathological features of the patients treated with Lenvatinib.

Pt (n°)	Sex (F/M)	Tumor histotype, variant	Metastasis	ECOG status	Age at Lenvatinib start (years)	Lenvatinib starting dose (mg/day)	Follow-up (months)	PAI-18.1 (Yes/No)	PAI-18.1 diagnosis after Lenvatinib started (months)	PAI-14 (Yes/No)	PAI-14 diagnosis after Lenvatinib started (months)
1	F	PTC, classic	Lung, neck	0	63	14	72	No	/	No	/
2	M	Hürtle cell	Lymph nodes, lung	0	44	24	94	No	/	No	/
3	F	PTC, solid	Lymph nodes, lung	0	40	24	75	Yes	15	Yes	21
4	M	FTC	Lung, neck, bone	0	77	20	62	Yes	3	Yes	6
5	M	FTC	Lymph nodes, lung	1	79	24	70	Yes	33	No	/
6	F	PTC, classic	Lung, bone	2	75	14	31	Yes	18	Yes	27
7	F	PTC, classic	Lymph nodes, lumg	1	82	10	71	No	/	No	/
8	F	PTC, tall cell	Lymph nodes, lung	1	67	24	82	Yes	3	Yes	15
9	M	PTC, tall cell	Bone, neck	0	73	24	44	Yes	9	Yes	9
10	F	PTC, classic	Lung, bone	1	61	24	97	Yes	15	Yes	27
11	F	PTC, solid	Lymph nodes, lung, bone	0	68	24	97	Yes	9	Yes	9
12	F	PTC, follicular	Lymph nodes, lung	1	66	24	26	Yes	12	Yes	21
13	M	PTC, tall cell	Lymph nodes, lung, bone, brain	0	56	10	9	No	/	No	/
14	F	Hürtle cell	Neck, lymph nodes	0	69	10	66	Yes	27	Yes	27
15	M	PTC, classic	Lymph nodes, lung	0	69	14	64	Yes	3	Yes	6
16	F	FTC	Lymph nodes, bone, kidney	1	64	10	30	Yes	9	Yes	9
17	M	FTC + PDTC	Lymph nodes, lung, bone	1	86	4	11	No	/	No	/
18	M	PTC, classic	Neck, lung, kidney	0	62	24	8	Yes	6	Yes	6
19	M	PTC, classxic	Lymph nodes, lung, bone	0	78	4	45	Yes	12	Yes	24
20	M	PTC + PDTC, trabecular, insular	Bone, lung	1	70	14	17	Yes	3	Yes	15
21	M	PTC, classic	Bone, lung	0	53	14	50	Yes	30	No	/
22	M	PTC, hobnail	Lymph nodes, lung, pleura	0	74	14	30	Yes	15	No	/
23	M	PDTC	Lung, neck	0	67	14	15	No	/	No	/
24	F	Hürtle cell	Neck, lymph nodes, lung, bone	0	73	14	9	Yes	3	No	/
25	F	PTC, classic	Neck, lymph node, lung	0	74	14	22	Yes	6	No	/
26	M	PTC, classic	Neck, lung, bone	0	71	10	48	No	/	No	/
27	F	PTC, classic	Lymph nodes, pleura	0	65	20	6	Yes	3	Yes	3
28	M	FTC	Neck, bone, lung	0	66	20	22	Yes	18	No	/
29	F	Hürtle cell	Neck, lung	0	66	10	22	Yes	21	No	/
30	F	PTC, classic	Lung, lymph nodes	1	70	10	6	Yes	6	No	/
31	M	PTC, classic	Lung, bone	0	65	14	19	Yes	3	Yes	9

ECOG, Eastern Cooperative Oncology Group; F, Female; FTC, follicular thyroid carcinoma; M, male; PAI, primary adrenal insufficiency; PDTC, poorly differentiated thyroid carcinoma; PTC, papillary thyroid carcinoma.

Starting dose of Lenvatinib was 16.06±6.2 mg (range 4–24 mg). Length of follow-up for the 31 patients was 42.58±29.53 months (range 6–97 months) (**Table 1**).

Before the beginning and during Lenvatinib treatment, all our patients had electrolytes, hemoglobin and renal function in the normal range. Moreover, there were no significant alterations of glycemia and hepatic function.

TSH levels were 0.21±0.24 µIU/ml at the enrollment. During Lenvatinib treatment, L-thyroxine dosage was titrated to maintain TSH values under 0.5 µIU/ml.

### Primary adrenal insufficiency

**Figure 1:**
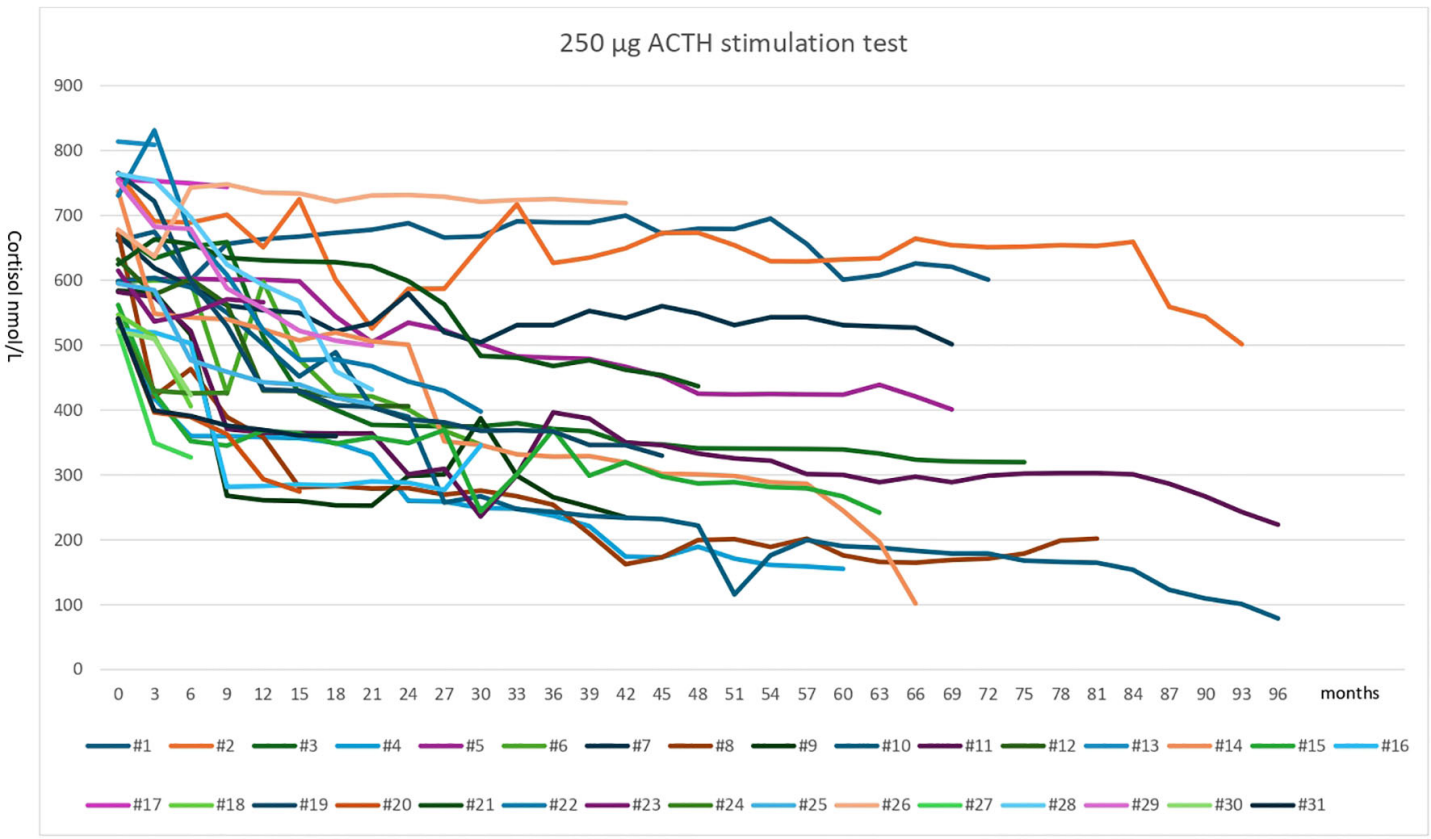
ACTH (250μg) stimulation test of all 31 patients treated with Lenvatnib at baseline (0, before starting therapy) and during follow-up. Patients with a subnormal response (peak cortisol < 18.1mcg/dL or 500nmol/L) started CA replacement treatment Moreover, the ACTH stimulation test was repeated after 3-6 months, stopping CA for 72 hours before testing, Further ACTH stimulation was repeated after 6-12 months in those patients feasible; in all cases adrenal insufficiency was confirmed. The peak of cortisol after ACTH stimulation test had a significant trend to decrease during Lenvatinib treatment (p=0.0011).

None of the 31 patients treated with Lenvatinib had PAI before starting treatment: in all cases the peak of cortisol was higher than 500 nmol/L or 18.1 mcg/dL (**Table 2**, **Figure 1**). The mean peak of cortisol was 635.6±90.26 nmol/L (M±SD) (range 521.4–813 nmol/L) before starting Lenvatinib treatment.

**Table 2:** Adrenal function before and during Lenvatinib treatment.

Pt (n°)/Sex	Cortisol peak before lenvatinib (nmol/L)	ACTH before lenvatinib (pg/ml)	PAI-18.1 (Yes/No)	PAI-18.1 diagnosis after lenvatinib started (months)	Cortisol peak at PAI-18.1 diagnosis (nmol/L)	ACTH at PAI diagnosis (pg/ml)	PAI-14 (Yes/No)	PAI-14 diagnosis after lenvatinib started (months)	Cortisol peak at PAI-14 diagnosis (nmol/L)	ACTH at PAI diagnosis (pg/ml)
1/F	661,2	16,2	No	/	/	/	No	/	/	/
2/M	765	21,1	No	/	/	/	No	/	/	/
3/F	670,8	11,9	Yes	15	426,1	53,5	Yes	21	377,4	67,8
4/M	539,9	26,7	Yes	3	418	56,8	Yes	6	360.2	71,8
5/M	596,6	39,4	Yes	33	483	72,9	No	/	/	/
6/F	598	25,2	Yes	9	425,9	115,3	Yes	27	368,2	118,2
7/F	669,1	34	No	/	/	/	No	/	/	/
8/F	672,5	26,4	Yes	3	421	31,5	Yes	15	281,1	77,4
9/M	584,1	39	Yes	9	268	95,7	Yes	9	268	95,7
10/F	599	36,5	Yes	15	452	65,3	Yes	27	257,7	91,9
11/F	582	34,2	Yes	9	371	91	Yes	9	371	91
12/F	632	35,3	Yes	12	430	197,1	No	/	/	/
13/M	813,8	42	No	/	/	/	No	/	/	/
14/F	736,8	37	Yes	27	351,7	126	Yes	27	351,7	126
15/M	562	11,3	Yes	3	425,6	21,2	Yes	6	352,1	35,8
16/F	523	18,7	Yes	9	281,6	95,9	Yes	9	281,6	95,9
17/M	755	27	No	/	/	/	No	/	/	/
18/M	547	16	Yes	6	406	51,6	No	/	/	/
19/M	765,2	19	Yes	12	432	58,4	Yes	24	385,7	63,2
20/M	541	38	Yes	3	396,3	111,5	Yes	15	274,6	113,5
21/M	624,9	18,3	Yes	30	483,7	49,8	No	/	/	/
22/M	731	8,6	Yes	15	477,4	51,3	No	/	/	/
23/M	614,8	20,5	No	/	/	/	No	/	/	/
24/F	534	24,7	Yes	3	429,8	91,1	No	/	/	/
25/F	595	18,7	Yes	6	476,9	167,2	No	/	/	/
26/M	678	11,5	No	/	/	/	No	/	/	/
27/F	522	42,8	Yes	3	349,3	93,8	Yes	3	349,3	93,8
28/M	763,8	7,2	Yes	18	459,8	15,8	No	/	/	/
29/F	752	11,4	Yes	21	499,3	27	No	/	/	/
30/F	521,4	28,4	Yes	6	423,1	173,2	No	/	/	/
31/M	540,8	10,5	Yes	3	399,2	72,4	Yes	9	375,9	72,4

F, Female; M, male; PAI, primary adrenal insufficiency.

#### Cut-off 500 nmol/L or 18.1 mcg/dL (PAI-18.1)

During Lenvatinib treatment, 24 of 31 patients (77.4%) had an insufficient response (peak cortisol <18.1 mcg/dL or 500 nmol/L) to ACTH stimulation (**Table 2**, **Figure 1**), supporting the diagnosis of adrenal insufficiency, according to current Endocrine Society Clinical Practice Guideline (11). In these 24 patients, peak cortisol at the moment of PAI-18.1 diagnosis was 414.3±60.69 nmol/L (M±SD), range 268–499 nmol/L.

13 patients with PAI-18.1 were female (54.2%) and 11 were male (45.8%) (**Table 2**). There was no association between sex and PAI-18.1 development.

The mean time of the development of PAI-18.1 was 11.75±9.01 months (range 3–33 months, median 9 months). In particular, during Lenvatinib therapy, PAI-18.1 had developed in 7 patients after three months, in 3 patients after six months, in 3 patients after nine months, in 2 patients after twelve months, in 3 patients after fifteen months, in 2 patients after eighteen months, in 1 patient after twenty-one months, in 1 patient after twenty-seven months, in 1 patient after thirty months and in 1 patient after thirty-three months. As consequence, 15 of the 24 (62.5%) patients developed PAI-18.1 within twelve months of the start Lenvatinib treatment (**Table 2**, **Figure 1**).

Patients with a subnormal response (peak cortisol < 18.1 mcg/dL or 500 nmol/L) started CA replacement treatment. Moreover, the ACTH stimulation test was repeated after 3–6 months, stopping CA for 72 hours before testing. Further ACTH stimulation was repeated after 6–12 months in those patients when feasible; in all cases adrenal insufficiency diagnosis was confirmed.

The peak of cortisol after ACTH stimulation test had a significant trend to decrease during Lenvatinib treatment (p= 0.0011) (**Figure 1**).

After one year of treatment, Lenvatinib was interrupted in a 76-year-old patient with PAI-18.1 for 34 days. The reason for this long interruption was due to surgical complications from laparoscopic cholecystectomy. In this patient, we repeated ACTH stimulation test after an appropriate interruption of CA; cortisol peak levels were normal (597.9 nmol/L); therefore, CA therapy was stopped and Lenvatinib restarted at the same dosage (14 mg) taken before laparoscopic cholecystectomy. However, after three months of Lenvatinib, two consecutive ACTH stimulation demonstrated a new development of PAI-18.1 (cortisol peak <500 nmol/L, twice).

There were not significant differences in the length of follow-up of patients who developed PAI-18.1 from those without PAI-18.1 (41.67±28.68 months vs 45.71±34.55 months; p=0.7556).

**Figure 2:**
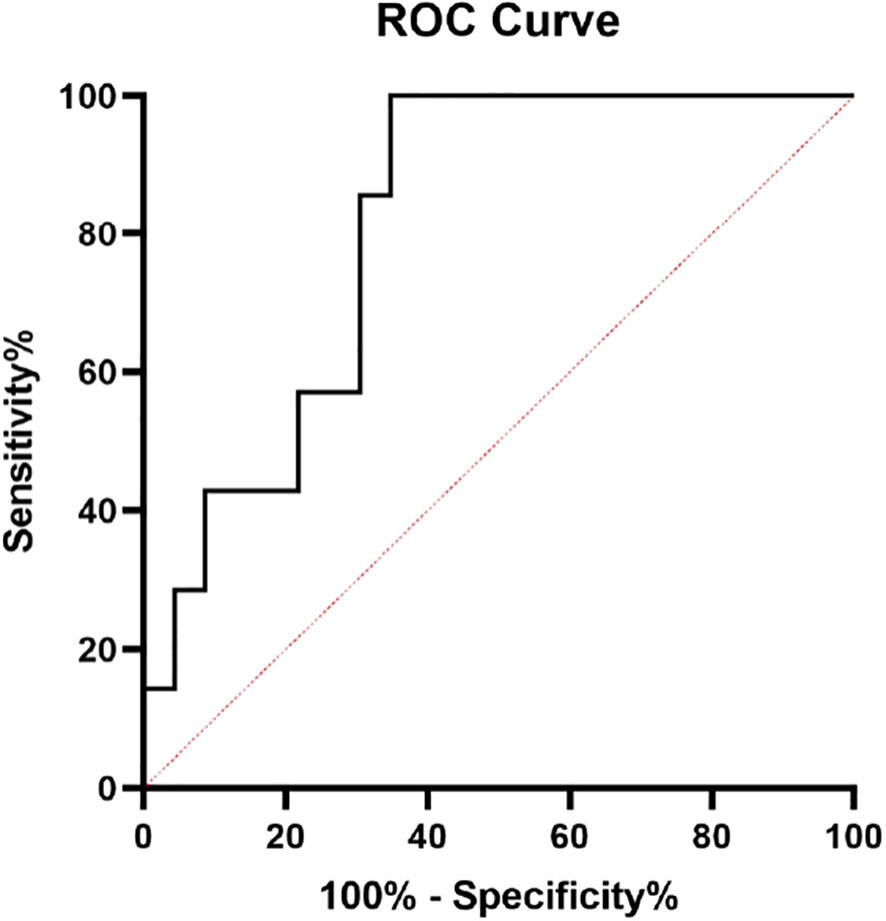
receiver operating characteristics(ROC) curve analysis to identify a peak cortisol cutoff to predict PAI development during Lenvatinib treatment. ROC curve analysis showed 646.6 nmol/L as the most accurate cutoff with a sensitivity of 69.57% (95% CI 49.13-84.4) and a specificity of 85.71% (48.69-99.27) [area under the curve (AUC) 0.8137, 95% CI 0.6591-0.9683, P=0.0133].

Before starting Lenvatinib treatment, the mean peak of cortisol after the first ACTH stimulation test was significantly lower in PAI-18.1 than in NOPAI-18.1 group (613.5±84.81 vs 708.1±70.64; p=0.0123). Considering this observation, we assessed the presence of a possible cut-off of cortisol peak to predict development of PAI-18.1 during Lenvatinib treatment. ROC curve analysis showed 646,6 nmol/L as the most accurate threshold with 69.57% sensitivity (95% CI 49.13-84.4) and 85.71% specificity (48.69-99.27) [area under curve (AUC) 0.8137, 95% CI 0.6591-0.9683, P = 0.0133] (**Figure 2**).

Using this cut-off, only 7 of the 24 patients with cortisol peak > 646.6 nmol/L developed PAI-18.1, while 17 out of the 24 patients with <646.6 nmol/L peak developed PAI-18.1 [p=0.0124; Odds ratio 14.57; Sensitivity 70.83%, Specificity 85.71%, Positive Predictive Value (PPV) 94.44%, Negative Predictive Value (NPV) 46.15%].

**Figure 3:**
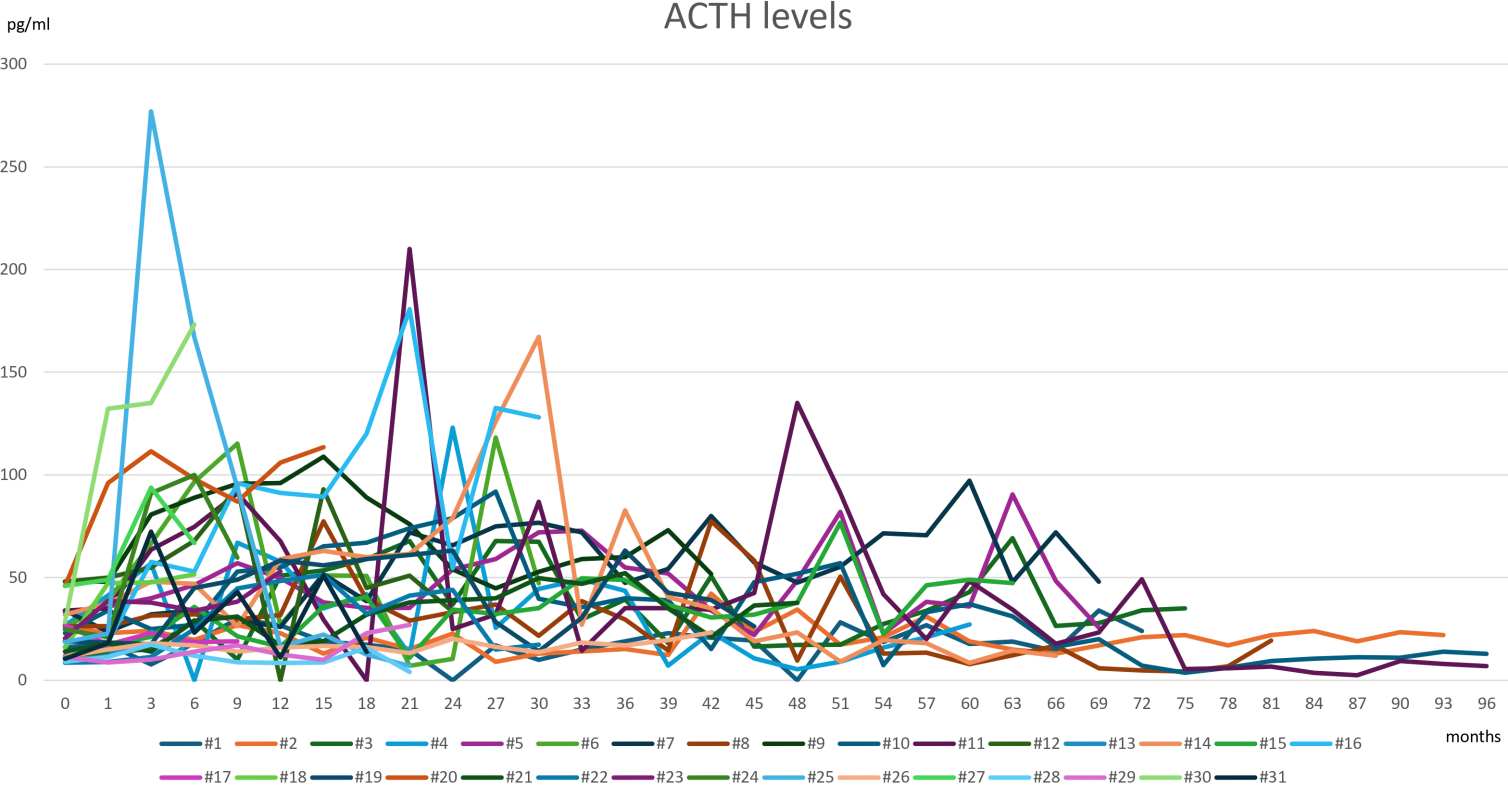
ACTH levels in all 31 patients treated with Lenvatanib at baseline (0, before starting therapy) and during follow-up (1-96 months).

Before treatment with Lenvatinib, ACTH levels were normal in all patients, with a mean±SD of 24.44±10.98 pg/ml (range 7.2–48.8 pg/ml). A progressive tendency to increasing ACTH levels was observed during treatment with Lenvatinib, although not statistically significant (**Figure 3**).

At the time of diagnosis of PAI-18.1, ACTH levels were significantly elevated compared to baseline values (83.97±46.6 pg/ml vs 24.38±11.32 pg/ml, p<0.0001).

PAI-18.1 diagnosis was associated with an increase of ACTH levels that often was observed months before the diagnosis. In particular, 15 out of 24 patients (62.5%) with PAI-18.1 had an increase of ACTH levels above reference values (>48.8 pg/ml) about 6.28±5.69 months (range 2–24 months) before PAI-18.1 diagnosis. In 6 out of 24 PAI-18.1 patients ACTH increase was concomitant with the diagnosis of PAI-18.1. In 3 out of 24 patients with PAI-18.1, an increase in ACTH occurred 6–12 months after the diagnosis of PAI-18.1. In these 3 patients without an increase of ACTH levels before or at the time of PAI-18.1 diagnosis we excluded a pituitary origin of adrenal insufficiency by evaluating other pituitary hormones and by pituitary magnetic resonance.

#### Cut-off 386.2 nmol/L or 14 mcg/dl (PAI-14)

We also considered a peak cortisol threshold of 386.2 mmol/L or 14 mcg/dL following ACTH stimulation testing. During Lenvatinib treatment, 14 out of 31 patients (45.2%) had an insufficient adrenal response (peak cortisol <14 mcg/dL or 386.2 nmol/L) and therefore a diagnosis of PAI-14. Of these, 8 were female (57.1%) and 6 were male (42.9%).

The mean time to development of PAI-14 was 14.79±8.76 months (range 3–27 months, median 12 months). In particular, during Lenvatinib therapy, PAI-14 has developed in 1 patient after three months, in 2 patients after six months, in 4 patients after nine months, in 2 patients after fifteen months, in 1 patient after twenty-one months, in 1 patient after twenty-four months and in 3 patients after twenty-seven months. Consequently, 7 out of the 14 (50%) patients developed PAI-14 within the first twelve months of Lenvatinib treatment.

There were no significant differences in follow-up between patients who developed PAI-14 and those who did not (52.5±29.59 months vs 34.41±27.69 months; p=0.0898), although the PAI-14 group had a longer follow-up.

The mean peak cortisol level after the first ACTH stimulation test, performed before starting Lenvatinib treatment, was significantly lower in the PAI-14 group compared to the NOPAI-14 group (591.9±62.98 vs 664.4±92.02; p=0.0195).

Using the same PAI-18.1 cut-off (ROC curve analysis), peak cortisol on ACTH stimulation before Lenvatinib, only 4 out of 14 patients with a cortisol peak >646.6 nmol/L developed PAI-14, while 10 out of 14 patients with a cortisol peak <646.6 nmol/L developed PAI-14 [p=0.0122; Odds ratio 8.125; Sensitivity 71.43%, Specificity 76.47%, Positive Predictive Value (PPV) 71.43%, Negative Predictive Value (NPV) 76.47%].

At the time of PAI-14 diagnosis, ACTH levels were significantly elevated compared to baseline values (86.6±23.9 pg/ml vs 26.94±11.23 pg/ml, p<0.0001). The diagnosis of PAI-14 was associated to an increase of ACTH levels that was observed several months before the formal diagnosis. Specifically, 13 out of 14 patients (92.8%) with PAI-14 showed elevated ACTH levels above the upper limit of normal (>48.8 pg/ml) approximately 11.77±8.38 months prior to PAI-14 diagnosis (range 2–27 months). In contrast, one patient exhibited an ACTH increase only 6 months after the diagnosis of PAI-14.

In all patients with PAI, adrenal metastases or other adrenal gland alterations were excluded by whole body computed tomography (CT) scans and whole body [18F]-fluoro-2-deoxy-D-glucose (FDG)-positron emission tomography/computed tomography (PET/CT) performed for tumor assessment.

Development of PAI-18.1 and PAI-14 was not associated to initial weight, follow-up duration, ECOG, sex, starting dose of Lenvatinib, dose intensity of Lenvatinib at the moment of PAI diagnosis, serum ACTH and cortisol levels before Lenvatinib treatment.

### Fatigue

During Lenvatinib treatment, fatigue was observed in 28 of 31 enrolled patients (90.3%).

Fatigue was not associated to initial weight, ECOG, sex, follow-up duration, serum ACTH and cortisol levels before Lenvatinib treatment, starting dose of Lenvatinib and dose intensity of Lenvatinib at the time of PAI diagnosis.

The mean time to the first onset of fatigue was 5.14 ± 9.35 months (range 1–45), while the mean time to reach peak fatigue severity was 13.63 ± 13.04 months (range 1–45). During Lenvatinib treatment, fatigue was observed in 23 of 24 patients with PAI-18.1 (95.8%) (**Table 3**), in 5 of 7 patients without PAI-18.1 (71.4%), in all 14 patients with PAI-14 (100%) (**Table 3**), and in 14 of 17 patients without PAI-14 (82.3%).

**Table 3:** Fatigue in patients with PAI: effect of treatment with cortisone acetate (CA).

Pt (n°)	Age at Lenvatinib start (years)	Follow-up (months)	Severity of fatigue Baseline (before Lenvatinib)	Severity of fatigue At the beginning of therapy with CA	Severity of fatigue 3 months after CA	Severity of fatigue 6 months after CA	Severity of fatigue Last follow-up (CA treatment)	PAI-14 (Yes/No)	Improvement of fatigue during CA treatment
3	40	75	0	2	1	1	1	Yes	Yes
4	77	62	0	2	1	1	1	Yes	Yes
5	79	70	0	3	2	2	2	No	Yes
6	75	31	0	2	1	1	1	Yes	Yes
8	67	82	0	3	2	2	3	Yes	Partial
9	73	44	0	2	1	1	1	Yes	Yes
10	61	97	0	2	1	1	3	Yes	Partial
11	68	97	0	2	0	0	1	Yes	Yes
12	66	26	0	2	2	2	2	No	No
14	69	66	0	2	1	0	1	Yes	Yes
15	69	64	0	2	1	1	2	Yes	Partial
16	64	30	1	3	3	3	2	Yes	Yes
18	62	8	0	3	2	1	1	No	Yes
19	78	45	0	3	1	1	2	Yes	Yes
20	70	17	0	3	1	1	1	Yes	Yes
21	53	50	0	1	1	1	1	No	No
22	74	30	0	0	0	0	0	No	/
24	73	9	0	1	2	2	/	No	No
25	74	22	1	3	2	2	2	No	Yes
27	65	6	0	2	1	/	/	Yes	Yes
28	66	22	0	3	2	/	/	No	Yes
29	66	22	1	3	/	/	/	No	/
30	70	6	0	2	/	/	/	No	/
31	65	19	0	2	1	1	1	Yes	Yes

**Table 3**. All patients in this table have a diagnosis of PAI-18.1. Fatigue was evaluated by the National Cancer Institute Common Terminology Criteria for Adverse Events (CTCAE) version 5.0 (11). Fatigue is defined as a disorder characterized by a state of generalized weakness with a pronounced inability to summon sufficient energy to accomplish daily activities. According to CTCAE, three grades of severity of fatigue were identified: Grade 1, fatigue relieved by rest; Grade 2, fatigue not relieved by rest, limiting activities of daily living, such as preparing meals, shopping for groceries or clothes, using the telephone, and managing money; Grade 3, fatigue not relieved by rest, limiting simple activities of daily living, such as bathing, dressing and undressing, feeding self, using the toilet, taking medications, and bedridden for much of the day. CA, cortisone acetato.

CA treatment was initiated in all patients with PAI-18.1; however, only 21 of the 23 patients with PAI-18.1 and fatigue had a follow-up period of CA treatment exceeding 3 months, which was necessary to adequately evaluate the efficacy of glucocorticoid replacement therapy. Among these, three patients (14.3%) did not experience improvement in fatigue during CA treatment, while 18 patients (85.7%) showed some degree of fatigue improvement: 15 patients experienced steady improvement, whereas 3 showed improvement during the first 3–6 months of CA therapy, which was not maintained at the last follow-up (**Table 3**).

In the 14 patients with PAI-14, treatment with CA led to an improvement in fatigue in all cases (**Table 3**). In 11 patients, the improvement remained stable until the last follow-up, while in 3 patients, fatigue improved during the first 3–6 months of treatment but was not maintained at the last follow-up.

Treatment with CA resulted in a significant improvement in fatigue in patients with PAI, both PAI-18.1 and PAI-14 (p < 0.0001). This improvement was mainly observed during the first 3 months of CA treatment (p < 0.0001) but persisted at 6 months (p = 0.0001) and at the last follow-up (p = 0.0007) (**Table 3**).

## Discussion

Among the adverse events evaluated during Lenvatinib treatment, routine monitoring of adrenal function is not commonly performed in real life; however, recent evidence from relatively small series of patients with advanced thyroid cancer has suggested a potential impact of Lenvatinib on development of PAI and fatigue (5–9).

It is now recognized that PAI can occur with Lenvatinib treatment, but its clinical significance remains unclear. Consequently, PAI is not always investigated in patients receiving Lenvatinib, which may delay diagnosis.

PAI is a serious and potentially life-threatening condition, and its simultaneous occurrence in patients with RR-DTC could have a profoundly negative impact on the morbidity and mortality of those treated with Lenvatinib, by increasing drug-related toxicity. In this context, early diagnosis plays a crucial role.

In this prospective study, using the current Endocrine Society Clinical Practice Guideline (12), we observed a higher prevalence (77.4%) of PAI-18.1 in patients with peak cortisol levels below 500 nmol/L (18.1 mcg/dL) compared to previous reports. This is likely because we routinely monitor adrenal function with ACTH stimulation testing more frequently than other studies, which often assess adrenal function only when ACTH levels are elevated.

Our study design therefore allows early identification of mild PAI cases. Furthermore, repeated ACTH stimulation tests performed during follow-up consistently confirmed the diagnosis of PAI and demonstrated a progressive decline in cortisol response during Lenvatinib treatment.

The mechanisms underlying Lenvatinib-induced PAI remain unknown. Lenvatinib is a multitargeted tyrosine kinase inhibitor (TKI); notably, two of its targets—the vascular endothelial growth factor receptors (VEGFRs) and platelet-derived growth factor receptor alpha (PDGFRα)—may be involved in adrenal regulation (14, 15). A key role seems to be played by the reduction of vascular density, mainly due to VEGF inhibition, causing ischemic damage to the adrenal cortex. Interestingly, a study in mice showed recovery of vascular damage following Lenvatinib withdrawal (16).

This experimental study supports the possibility that the adverse effect of Lenvatinib on adrenal function may be transient and dose-dependent, with recovery of adrenal function following drug discontinuation, as is common with other treatment-related adverse events. Notably, in one of our patients with PAI, Lenvatinib was interrupted for 34 days after one year of treatment due to surgical complications following laparoscopic cholecystectomy. After appropriate discontinuation of cortisone acetate, repeated ACTH stimulation test showed normal peak cortisol levels (597.9 nmol/L). Lenvatinib was then resumed at the previous dose (14 mg). However, after three months of reinitiation, two consecutive ACTH stimulation tests revealed a recurrence of PAI.

The strength of our study is the ability to evaluate adrenal function both before and during treatment with Lenvatinib. All our patients had normal adrenal function prior to starting Lenvatinib. Moreover, none of the patients presented with adrenal metastases or other adrenal gland abnormalities that could cause PAI, as confirmed by whole-body CT scans and whole-body 18F-FDG PET/CT during follow-up. Together, these findings support the conclusion that PAI is induced by Lenvatinib.

In recent years, there has been a proposal to lower the serum cortisol cut-off for the diagnosis of primary adrenal insufficiency (PAI) compared to the threshold established in the 2016 Endocrine Society Clinical Practice Guideline (12). While using an ACTH-stimulated peak cortisol threshold of 18 mcg/dL (500 nmol/L) to exclude adrenal insufficiency minimizes missed diagnoses, it may lead to overdiagnosis when more specific assays are employed. Consequently, several recent studies utilizing more specific (less cross-reactive) cortisol assays have suggested lowering the ACTH-stimulated peak cortisol cut-off to between 14 mcg/dL (386.2 nmol/L) and 15 mcg/dL (413.8 nmol/L) to reduce false-positive results in ACTH stimulation tests (13, 17).

In this context, we applied an ACTH-stimulated peak cortisol cut-off of 14 mcg/dL as a diagnostic criterion for PAI (PAI-14). As expected, the prevalence of PAI-14 was lower than that of PAI-18.1; however, it remained substantial, with 14 out of 31 patients (45.2%) meeting the PAI-14 criterion.

The diagnosis of PAI-14 was preceded, on average, by an increase in ACTH levels 11.77 ± 8.38 months earlier in 92.3% of cases. This rise was greater, although not statistically significant (p = 0.054), compared to the increase observed before the diagnosis of PAI-18.1, which occurred in only 62.5% of cases and on average 6.28 ± 5.69 months earlier.

This observation indicates that cortisol deficiency is, as expected, more pronounced in PAI-14 than in PAI-18.1. Indeed, cortisol deficiency leads to reduced negative feedback on the hypothalamic-pituitary axis and a consequent compensatory increase in ACTH levels, reflecting greater biochemical and clinical impairment of PAI (12).

Treatment with cortisone acetate (CA) resulted in a significant improvement in fatigue, particularly during the first 3 months of therapy, with benefits persisting in the majority of cases, especially among patients with PAI-14.

Fatigue is one of the most common treatment-related adverse events, with a reported prevalence of up to 90–100% of cases (18–22). The wide variability in fatigue prevalence reported in the literature is partly due to the frequent underestimation and underdiagnosis of fatigue in clinical practice, despite it being a distressing and disabling condition.

Fatigue is a major cause of dose reduction, interruption, or discontinuation of Lenvatinib treatment, potentially leading to decreased treatment efficacy and shorter progression-free survival (PFS) (4). Managing fatigue effectively is critical to maintain optimal treatment adherence and maximize clinical benefit (23).

The mechanisms underlying fatigue are not yet fully elucidated. Fatigue arises from a complex interplay of multiple factors, including physical, psychological and biochemical conditions (24).

This study confirms that in the presence of fatigue, it is necessary to exclude PAI. If PAI is diagnosed, prompt initiation of glucocorticoid replacement therapy is essential to manage fatigue in Lenvatinib treatment.

Based on current evidence, we propose that all patients considered for Lenvatinib treatment undergo evaluation of adrenal function using a 250 μg ACTH stimulation test along with serum ACTH level measurement. A cortisol peak greater than 646.6 nmol/L indicates a low risk (approximately 30%) of developing PAI. In such cases, if ACTH levels are within the normal range, repeat ACTH stimulation testing is not mandatory; however, if ACTH levels are elevated beyond the upper limit of normal, ACTH stimulation testing should be repeated within 3 to 6 months.

Conversely, a cortisol peak below 646.6 nmol/L suggests a high risk (about 70%) of PAI development. For these patients, ACTH stimulation tests should be repeated according to ACTH concentrations: if ACTH is normal, retesting may be delayed but should occur within 6 to 12 months; if ACTH levels are elevated, repeat ACTH stimulation should be performed as soon as possible to guide timely diagnosis and management.

There are some limitations of our study that should be considered. It is a prospective single-center investigation conducted at a tertiary referral center, involving a relatively small sample size of 31 patients, which may limit the generalizability of the findings to broader populations of patients with RR-DTC treated with Lenvatinib. Additionally, the limited sample size may lack sufficient statistical power to detect weaker associations between PAI development, baseline clinical characteristics, and treatment parameters (e.g., initial dose, dose intensity, duration).

In conclusion, based on our experience and supported by the literature, we recommend that adrenal function assessment be incorporated into the evaluation of patients treated with Lenvatinib, especially those experiencing fatigue. When PAI is confirmed, initiating glucocorticoid replacement therapy is advisable to manage fatigue and prevent unnecessary dose reduction or discontinuation of Lenvatinib treatment. This approach aligns with findings that PAI is a common and treatable cause of fatigue in this patient population, and that early intervention improves clinical outcomes and quality of life.

## Data Availability

The raw data supporting the conclusions of this article will be made available by the authors, without undue reservation.
